# Intraoperative ventilation: incidence and risk factors for receiving large tidal volumes during general anesthesia

**DOI:** 10.1186/1471-2253-11-22

**Published:** 2011-11-21

**Authors:** Ana Fernandez-Bustamante, Cristina L Wood, Zung V Tran, Pierre Moine

**Affiliations:** 1 Department of Anesthesiology, University of Colorado, USA; 2 Department of Biostatistics and Informatics, University of Colorado, USA

## Abstract

**Background:**

There is a growing concern of the potential injurious role of ventilatory over-distention in patients without lung injury. No formal guidelines exist for intraoperative ventilation settings, but the use of tidal volumes (V_T_) under 10 mL/kg predicted body weight (PBW) has been recommended in healthy patients. We explored the incidence and risk factors for receiving large tidal volumes (V_T _> 10 mL/kg PBW).

**Methods:**

We performed a cross-sectional analysis of our prospectively collected perioperative electronic database for current intraoperative ventilation practices and risk factors for receiving large tidal volumes (V_T _> 10 mL/kg PBW). We included all adults undergoing prolonged (≥ 4 h) elective abdominal surgery and collected demographic, preoperative (comorbidities), intraoperative (i.e. ventilatory settings, fluid administration) and postoperative (outcomes) information. We compared patients receiving exhaled tidal volumes > 10 mL/kg PBW with those that received 8-10 or < 8 mL/kg PBW with univariate and logistic regression analyses.

**Results:**

Ventilatory settings were non-uniform in the 429 adults included in the analysis. 17.5% of all patients received V_T _> 10 mL/kg PBW. 34.0% of all obese patients (body mass index, BMI, ≥ 30), 51% of all patients with a height < 165 cm, and 34.6% of all female patients received V_T _> 10 mL/kg PBW.

**Conclusions:**

Ventilation with V_T _> 10 mL/kg PBW is still common, although poor correlation with PBW suggests it may be unintentional. BMI ≥ 30, female gender and height < 165 cm may predispose to receive large tidal volumes during general anesthesia. Further awareness of patients' height and PBW is needed to improve intraoperative ventilation practices. The impact on clinical outcome needs confirmation.

## Background

The lung can be injured by positive pressure ventilation. Mechanical stretch triggers a proinflammatory response within the first 2 hours in healthy animal models [1-4]. The benefit of lung protective ventilation (LPV) with low tidal volumes (V_T_), usually 6 mL/kg predicted body weight (PBW), has been strongly evidenced for patients with acute lung injury and acute respiratory distress syndrome (ALI/ARDS) [5-8]. LPV strategies, designed to limit end-inspiratory volumes and pressures, were associated with reduced inflammatory markers in bronchoalveolar lavage fluid and blood [6-8] and improved clinical outcomes [5,7,8].

In patients without evidence of existing lung injury, the significance of ventilator-induced lung injury is controversial [9-17]. Clinical studies favoring a LPV regimen in non-ALI patients suggest a decreased inflammatory or pro-coagulation mediators with LPV strategies compared to conventional ventilation [12,14], and some have found improvement in clinical outcomes after thoracic or esophageal surgery [11,13,15]. However, inevitable confounders in these studies such as different PEEP and oxygen fractions, and concerns of potential effects of LPV (i.e. atelectasis, hypercapnia, etc.) have prevented from reaching widespread application. Guidelines for intraoperative ventilation are lacking, and the recommended safety threshold for healthy patients has unofficially been set at V_T _< 10 mL/kg PBW [18-21].

We hypothesized that V_T _> 10 mL/kg PBW is still often applied in routine intraoperative ventilatory set up. We expected a < 10% of patients receiving unintentional large tidal volumes because of reduced height or obesity-related height/weight disproportion. We performed an observational cross-sectional analysis of our perioperative electronic database to study the incidence of and risk factors for receiving intraoperative V_T _> 10 mL/kg PBW during general anesthesia.

## Methods

After IRB exempt approval (COMIRB#10-0551), all patients ≥ 18 years old who underwent elective abdominal surgery of ≥ 4 h at our institution from August 2007 to May 2010 were included in this cross-sectional analysis. A threshold of 4 h duration was arbitrarily chosen to exclude short abdominal procedures where mechanical ventilation might be too brief and/or unreflective of non-spontaneous ventilatory settings. All data have been collected from clinical documentation entered by anesthesiology residents, attending staffs, and certified registered nurse anesthetists into the institution's perioperative clinical information system (Centricity^® ^General Electric Healthcare, Waukesha, WI). Collected information included patient characteristics (age, gender, height weight, preoperative comorbidities: obesity, defined as a body mass index, BMI, ≥ 30 kg/m^2^, asthma, COPD/emphysema, obstructive sleep apnea, oxygen dependency, congestive heart failure, diagnosed cancer), intraoperative management (ventilatory settings, blood gas analysis, administered intravenous fluids, need of blood products or vasopressors), and postoperative course and outcomes (need of postoperative mechanical ventilation (POMV) and ICU admission, duration of mechanical ventilation, ICU and hospital length of stay, in-hospital mortality).

Patients' predicted body weight was calculated by the following formulas [7]: Males: PBW (kg) = 50 + 0.91 × (height (cm) - 152.4); Females: PBW (kg) = 45.5 + 0.91 × (height (cm) - 152.4). Exhaled recorded tidal volumes (V_T_), in mL per kg PBW were calculated. Patients with median values of intraoperative exhaled V_T _less than 8 mL/kg PBW (< 8 mL/kg PBW), 8-10 mL/kg PBW (8-10 mL/kg PBW) and greater than 10 mL/kg PBW (> 10 mL/kg PBW) were a priori selected for comparison. While V_T _10 mL/kg PBW is the V_T _limit mostly accepted and recommended as safe in healthy patients, the V_T _< 8 mL/kg PBW threshold is an arbitrary limit chosen in this study for the purpose of statistical comparison. Though arbitrary, V_T _values < 8 mL/kg PBW are more similar than 8-10 mL/kg PBW to the resting tidal volumes of spontaneously breathing adults (7-8 mL/kg) and also to those V_T _values shown to be protective in ARDS patients [5,7]. All aforementioned variables (demographics, comorbidities, intraoperative management and outcomes) from the three V_T _subgroups were compared to detect differences that may be implicated in the use of V_T _> 10 mL/kg PBW.

### Statistical Analysis

Descriptively, for continuous variables, mean ± SD are shown, nominal variables are shown as percentages (n, %) [7]. Median values of ventilatory parameters (V_T_, respiratory rate, peak pressure, etc) from each patient were selected, instead of the means, for calculating the subgroups averages to minimize errors of extreme values from spontaneous ventilation or rapid intraoperative adjustments. Variables were compared using either one-way ANOVA or Chi-square to detect potential differences between the different V_T _subgroups: > 10 mL/kg PBW, 8-10 mL/kg PBW and < 8 mL/kg PBW. Logistic regression analysis was performed including patients from all V_T _subgroups with significant variables from the univariate analysis. All statistical analysis was performed with SPSS 18.0 software. p < 0.05 was considered to be statistically significant.

## Results

429 patients met inclusion criteria and their characteristics are summarized in Table 1. Exhaled V_T _values from all patients averaged 579.0 ± 99.3 mL (8.7 ± 1.6 mL/kg PBW) and ranged from 344.0 mL (5.1 mL/kg PBW) to 880.0 mL (15.4 mL/kg PBW). 154 patients (35.9%) were ventilated with a median exhaled V_T _< 8 mL/kg PBW, 200 patients (46.6%) with a V_T _8-10 mL/kg PBW, and 75 patients (17.5%) with V_T _> 10 mL/kg PBW. Volume Control Ventilation was used in 386 (90.0%) patients, Pressure Control Ventilation in 4 (0.8%) patients. The ventilatory mode was not recorded in 39 (9.1%) patients.

**Table 1:** Demographic characteristics of patients who underwent prolonged (≥ 4 h) abdominal surgery.

Number	429
Age (years), Mean ± SD	58.1 ± 14.9

Gender distribution	
Male, n(%)	293(68.3%)
Female, n(%)	136(31.7%)

ASA classification	
1, n(%)	21(4.9%)
2, n(%)	169(39.4%)
3, n(%)	194(45.2%)
4, n(%)	13(3.0%)
Unclassified	32(7.5%)

Comorbidities	
COPD, n(%)	30(7.0%)
Asthma, n(%)	30(7.0%)
Obstructive Sleep Apnea, n(%)	63(14.7%)
Oxygen dependency, n(%)	18(4.2%)
Obesity (BMI ≥ 30), n(%)	147(34.3%)
Congestive Heart Failure, n(%)	14(3.3%)
Cancer, n(%)	276(64.3%)

Surgical diagnosis	
Cancer (GI, GYN or GU origin), n(%)	276(64.3%)
GU stricture/fistula repair, n(%)	78(18.2%)
Incontinence/Neurogenic bladder, n(%)	36(8.4%)
Other, n(%)	39(9.1%)

Duration of surgical procedure (minutes), Mean ± SD	347.5 ± 94.6

Outcomes	
POMV and ICU admission, n(%)	62(14.5%)
POMV duration (days), Mean ± SD^a^	1.8 ± 3.4
ICU LOS (days), Mean ± SD^a^	4.1 ± 5.7
Hospital LOS (days), Mean ± SD	7.0 ± 6.7
In-hospital mortality, n(%)	6(1.4%)

(ASA = American Society of Anesthesiologists; BMI = Body Mass Index; COPD = Chronic Obstructive Pulmonary Disease; GI = Gastrointestinal; GYN = Gynecological; GU = Genitourinary; POMV = Postoperative mechanical ventilation)

^a ^Only patients that required POMV and ICU stay were included in this calculation

Statistically significant differences between V_T _subgroups were found in several variables (Table 2). When patients from both extreme subgroups were compared, patients in the V_T _> 10 mL/kg PBW subgroup showed a significantly greater proportion of females and obese patients (defined as BMI ≥ 30) than patients receiving V_T _< 8 mL/kg PBW_. _Patients in the V_T _> 10 mL/kg PBW had significantly smaller heights and predicted body weights (PBW), although the average weight was similar and consequently the average body mass index (BMI) was significantly greater than those receiving V_T _< 8 mL/kg PBW. The larger median exhaled V_T _was associated with a significantly increased minute volume ventilation, reduced respiratory rates, greater peak pressures and lower values of end-tidal CO_2 _partial pressure. No differences in PEEP pressure, inspired oxygen fraction or arterial oxygenation were found between V_T _subgroups. Duration of the surgical procedure and the estimated blood loss were slightly greater in the V_T _> 10 mL/kg PBW subgroup but did not reach statistical significance. Transfusion of blood products, but not of other intravenous fluids, was significantly increased in the large V_T _subgroup.

**Table 2:** Characteristics of patients according to their intraoperative median exhaled tidal volumes (V_T_) (mL/kg PBW).

	Median V_T _subgroups (mL/kg PBW)^a^			
	**< 8**	**8-10**	**> 10**	**p value**

Number, n(%)	154(29.5%)	200(38.3%)	75(14.4%)	

Age (years), Mean ± SD	59.6 ± 14.1	57.4 ± 15.1	56.9 ± 15.6	0.298

Gender distribution				≤ 0.001
Male, n(%)	124(80.5%)	141(70.5%)	28(37.7%) *	
Female, n(%)	30(19.5%)	59(29.5%)	47(62.7%) *	

Height (cm), Mean ± SD	177.9 ± 9.7	173.7 ± 9.7	161.4 ± 11.0 *	≤ 0.001
Weight (kg), Mean ± SD	84.1 ± 20.2	86.3 ± 21.3	89.6 ± 25.9	0.207
Predicted Body Weight (PBW)(kg), Mean ± SD	72.9 ± 9.9	68.4 ± 10.2	55.7 ± 10.8 *	≤ 0.001
Body Mass Index (BMI), Mean ± SD	26.5 ± 5.4	28.4 ± 5.4	34.1 ± 8.9 *	≤ 0.001

ASA classification				0.884
1, n(%)	6(3.9%)	12(6.0%)	3(4.0%)	
2, n(%)	61(39.6%)	79(39.5%)	29(38.7%)	
3, n(%)	73(47.4%)	86(43.0%)	35(46.7%)	
4, n(%)	6(3.9%)	5(2.5%)	2(2.6%)	
Unclassified, n(%)	8(5.2%)	18(9.0%)	6(8.0%)	

Comorbidities				
COPD, n(%)	12(7.8%)	16(8.0%)	2(2.7%)	0.270
Asthma, n(%)	10(6.5%)	14(7.0%)	6(8.0%)	0.916
Obstructive Sleep Apnea, n(%)	18(11.7%)	32(16.0%)	13(17.3%)	0.407
Oxygen dependency, n(%)	8(5.2%)	7(3.5%)	3(4.0%)	0.730
Obesity (BMI ≥ 30), n(%)	33(22.6%)	64(33.2%)	50(68.5%) *	≤ 0.001
Congestive Heart Failure, n(%)	2(1.3%)	11(5.5%)	1(1.3%)	0.051
Cancer, n(%)	106(68.8%)	124(62.0%)	46(61.3%)	0.681

Intraoperative ventilatory management				
Minute ventilation (L/min), Mean ± SD	75.9 ± 14.2	88.5 ± 13.8	108.7 ± 21.5 *	≤ 0.001
Respiratory rate (breath/min), Mean ± SD	10.6 ± 1.9	10.0 ± 1.6	9.6 ± 1.6 *	≤ 0.001
Peak pressure (cmH_2_O), Mean ± SD	20.9 ± 6.3	22.2 ± 5.6	23.6 ± 6.6 *	0.004
PEEP (cmH_2_O), Mean ± SD	3.7 ± 1.9	3.8 ± 2.3	3.9 ± 1.9	0.819
End-tidal CO_2 _(mmHg), Mean ± SD	35.4 ± 4.2	34.3 ± 2.6	33.9 ± 2.7 *	0.004
Mean FiO_2_, Mean ± SD	70.2 ± 27.2	70.6 ± 26.6	74.0 ± 25.2	0.699
PaO_2_/FiO_2 _ratio, Mean ± SD	298.9 ± 188.2	315.2 ± 249.4	248.1 ± 157.5	0.196

Intraoperative use of vasopressors, n(%)	25(16.2%)	28(14.0%)	13(17.3%)	0.741

Intraoperative fluid management				
Infused crystalloids (mL/kg/h), Mean ± SD	7.8 ± 3.2	8.3 ± 3.6	8.6 ± 3.7	0.213
Infused colloids (mL/kg/h), Mean ± SD	1.8 ± 1.2	1.8 ± 1.6	1.4 ± 0.9	0.656
Estimated blood loss (mL), Mean ± SD	650.6 ± 661.3	749.6 ± 802.5	869.1 ± 887.8	0.127
Fluid balance (evaporation & insensible losses not included) (mL/kg/h), Mean ± SD	6.5 ± 3.3	7.1 ± 3.6	7.7 ± 4.1	0.079
Use of blood products, n(%)	27(17.5%)	55(27.5%)	27(36.0%) *	0.007

Duration of surgical procedure (minutes), Mean ± SD	340.8 ± 92.2	345.6 ± 94.4	366.0 ± 98.7	0.155

Outcomes				
POMV and ICU admission, n(%)	17(11.0%)	27(13.5%)	18(24.0%) *	0.028
POMV duration (days), Mean ± SD^b^	1.5 ± 2.8	1.9 ± 3.8	2.1 ± 3.7	0.801
ICU LOS (days), Mean ± SD^b^	3.3 ± 3.2	4.8 ± 6.4	3.7 ± 6.6	0.488
Hospital LOS (days), Mean ± SD	6.2 ± 6.1	7.0 ± 6.6	8.3 ± 7.8 *	0.101
In-hospital mortality, n(%)	3(1.9%)	2(1.0%)	1(1.3%)	0.752

(ASA = American Society of Anesthesiologists; BMI = Body Mass Index; FiO_2 _= Inspired fraction of oxygen; PaO_2 _= arterial oxygen partial pressure; PEEP = Positive End-Expiratory Pressure; POMV = Postoperative mechanical ventilation)

(p values shown in last column refer to comparison of the three groups, * refers to p < 0.05 in comparison of extreme groups)

^a ^Predicted body weight was calculated by the following formulas [7]: Males: PBW (kg) = 50 + 0.91 × (height (cm) - 152.4); Females: PBW (kg) = 45.5 + 0.91 × (height (cm) - 152.4)

^b ^Only patients that required POMV and ICU stay were included in this calculation

Logistic regression analysis, including all significant variables from the univariate analysis, found obesity as the most significant risk factor for a patient receiving a V_T _> 10 mL/kg PBW (OR 8.7) but did not result in a reliable prediction equation (overall correct classification 85.4%, Nagelkerke R^2 ^= 0.61). The use of blood products was not significant in this analysis.

**Figure 1:**
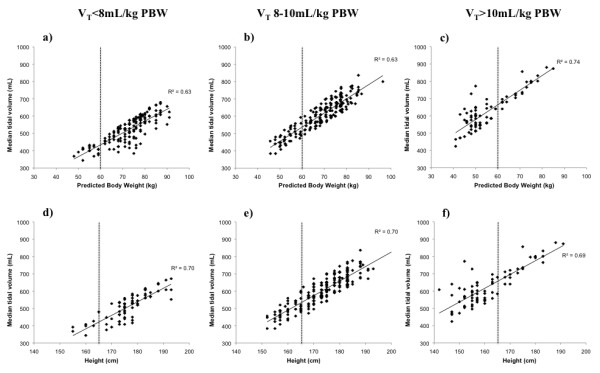
**Relationship between intraoperative median tidal volumes (V_T_) and patients' predicted body weight (PBW) (1.a-1.c) and height (1.d-1.f) in patients from all V_T _subgroups**. Note the PBW and height distribution in the different VT subgroups, with most patients in the V_T _> 10 mL/kg PBW subgroup having a PBW < 60 kg and height < 165 cm while the opposite was observed in the V_T _< 8 mL/kg PBW and a more uniform distribution was observed in the V_T _8-10 mL/kg PBW.

The relationship between the median exhaled V_T _(in mL) of all patients plotted against their actual body weight and predicted body weight (PBW) showed a poor correlation (correlation coefficients: 0.30 and 0.24, respectively). The correlation between the V_T _with the PBW was greater when patients from the V_T _subgroups were plotted separately (Figures 1a-c). Note that most patients in the V_T _< 8 mL/kg PBW had heights > 165 cm and PBW > 60 kg, while most patients in the V_T _> 10 mL/kg PBW had heights < 165 cm and PBW < 60 kg (Figures 1d-f).

**Figure 2:**
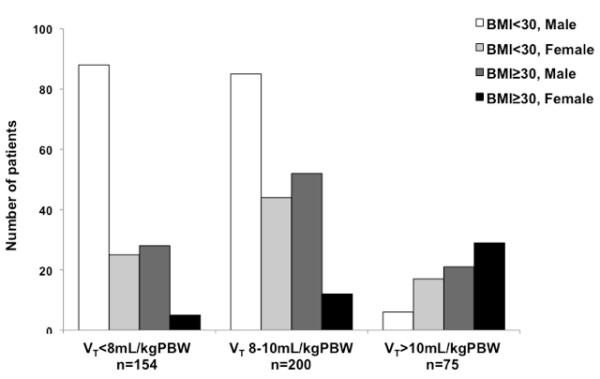
**Distribution of obesity and gender on the intraoperative tidal volume (V_T_) subgroups**. The distribution was significantly different between subgroups (p ≤ 0.001), with most patients in the < 8 mL/kg PBW being non-obese (73.3%) and males (75.3%) but obese (66.7%) and females (61.4%) in the > 10 mL/kg PBW subgroup. There was missing information from 8 patients in the < 8 mL/kg PBW subgroup, 7 patients in the 8-10 mL/kg PBW subgroup and 2 patients in the > 10 mL/kg PBW subgroup (5%, 3.5% and 2.7%, respectively) that precluded their classification.

34.0% of all obese patients and 34.6% of all females in the 429 patient cohort were ventilated using V_T _> 10 mL/kg PBW. Figure 2 shows the significantly different distribution of gender and obesity in the V_T _subgroups (p ≤ 0.001).

**Figure 3:**
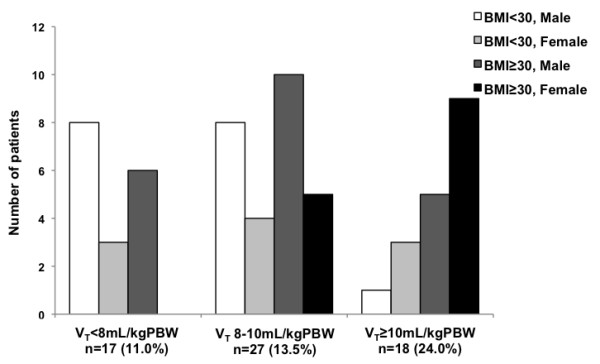
**Gender and obesity characteristics of patients that needed postoperative mechanical ventilation (POMV) and ICU admission in tidal volume (V_T_) subgroups**. The incidence of POMV was significantly different in the V_T _subgroup (V_T _> 10 mL/kg PBW n = 18, 24.0%) compared to the other subgroups (n = 17, 11.0%, in V_T _< 8 mL/kg PBW and n = 29, 13.5%, in the V_T _8-10 mL/kg PBW) (p = 0.028). The distribution of gender and obesity of these patients was also significantly different between V_T _subgroups (p = 0.022).

The incidence of postoperative mechanical ventilation (POMV) and ICU admission was greater in patients receiving V_T _> 10 mL/kg PBW compared with the V_T _< 8 mL/kg PBW subgroup. POMV duration and ICU length of stay were comparable in both groups, but hospital length of stay was more prolonged in the large V_T _subgroup. The distribution of gender and obesity in patients requiring POMV and ICU admission was significantly different in the V_T _subgroups (Figure 3). Of those 62 patients needing POMV and ICU admission, only 31 required mechanical ventilation for at least 24 h and had ventilatory data recorded. Pressure ventilatory modes were the only controlled or assisted/controlled modes used (PRVC, PCV, PSV, CPAP). Median tidal volumes in the ICU were significantly smaller than those used intraoperatively (573.9 ± 90.4 vs. 530.8 ± 98.6, n = 26) (p = 0.040 by paired t-test). 19 of these 31 patients had oxygenation criteria of ARDS (PaO_2_/FiO_2 _< 200) and 2 of ALI (PaO_2_/FiO_2 _200-300) (radiographic criteria not available) during their ICU stay: 7 patients had received an intraoperative V_T _< 8 mL/kg PBW (4.5% of that V_T _subgroup), 9 a V_T _8-10 mL/kg PBW (4.5% of that V_T _subgroup) and 5 a V_T _> 10 mL/kg PBW (6.7% of that V_T _subgroup) (p = 0.736). 6 patients died during their hospital postoperative stay. All of them had been admitted to the ICU, but their intraoperative ventilatory settings were not significantly different.

## Discussion

The present study showed that: 1) Intraoperative ventilatory settings are not uniform; 2) intraoperative ventilation with large tidal volumes (V_T _> 10 mL/kg PBW) may occur in more than 15% of surgical patients; 3) intraoperative tidal volumes do not routinely correlate with accurate predicted body weight calculations; and 4) obesity, female gender or short height are risk factors for receiving large V_T _during prolonged abdominal surgery.

Our study presents obvious limitations: it is a historical analysis that includes a relatively small number of patients from a single institution. Our results, however, suggest that the lack of formal guidelines on intraoperative ventilation practices is leading to a significant percentage of surgical patients receiving large tidal volumes.

Intraoperative ventilatory settings were not uniform in our study, reflected by the poor correlation between the tidal volumes in the whole patient sample with the predicted body weights or other size variable. The range of observed tidal volumes in mL/kg of predicted body weight was wider than expected, especially the highest tidal volume values.

Avoidance of large tidal volumes is, at present, the most efficient strategy to prevent and/or treat acute lung injury (ALI) or acute respiratory distress syndrome (ARDS) [5,7,22,23]. In patients without lung injury or risk factors for it, recent reviews recommend the use of V_T _< 10 mL/kg PBW [18,19]. Other authors have previously observed benefits of a low V_T _ventilation strategy in surgical patients without evidence of lung injury, in terms of decreased inflammation or improved outcomes [10-15,24]. In our study, one out of every 6 patients (17.5% of patients) received intraoperative V_T _> 10 mL/kg PBW. This incidence was greater than expected. However, we were not able to find any recent literature presenting the current ventilatory practices during general anesthesia without a predefined intervention or protocol. Furthermore, it is known that compliance with low V_T _ventilation strategies was also difficult to implement even in the ICU setting [25-28], where patients usually present one or more risk factors for ALI/ARDS. Resistance to applying lung protection ventilation strategies has also been observed in patients with ALI criteria during general anesthesia for surgical procedures [29]. So, although potentially a single institution problem, the incidence of V_T _> 10 mL/kg PBW may be an underestimated incident that needs to be confirmed and addressed. Measures have been implemented in our institution to minimize these suboptimum practices.

Predicted body weight calculation in adult patients is determined by the patient's gender and height [7]. The correlation between V_T _and the PBW in our patients presented a poor correlation. This correlation greatly improved when the tidal volume subgroups were studied separately, which suggests an important role of the wide range of V_T _used (344.0 to 880.0 mL, corresponding to 5.1 to 15.4 mL/kg PBW) on the scattering of the whole sample. However, despite a better correlation of V_T _with the PBW in each V_T _subgroup, the wide range of V_T _values still reflects the challenging and often erroneous height-based PBW estimate. Furthermore, PBW and height in the large V_T _subgroup tended to be "shifted to the left" compared to values in the lower V_T _subgroup (compare Figures 1a and 1d with 1c and 2f, respectively). This reflects, in our opinion, an inaccurate estimation of the patients' height and therefore PBW in those patients receiving V_T _> 10 mL/kg PBW. The PBW formula is not an easy mental calculation, and the lack of effect of weight or BMI is somehow counterintuitive to some providers. These two factors may explain why patients of shorter height and obese patients, with unusual height/weight proportions, are more likely affected by the unintentional use of large tidal volumes. The risk of females and/or short height for receiving large tidal volumes has been observed before in the ICU setting [30]. Nonetheless, the surprisingly high incidence in the intraoperative setting of large tidal volumes has, to our knowledge, not been reported. The fact that half of patients with a height < 165 cm, one third of patients with a BMI ≥ 30 or one third of females may be receiving these large tidal volumes during general anesthesia deserves, in our opinion, further awareness and creative solutions.

The significantly worse clinical outcomes (greater incidence of POMV and ICU admission and longer hospital stay) with the use of V_T _> 10 mL/kg PBW compared to the V_T _< 8 mL/kg PBW subgroup are difficult to explain. Known predictors of poor outcome are likely to have been involved in the greater incidence of POMV and ICU admission in the high V_T _subgroup, including obesity and other preoperative comorbidities [31-33] and intraoperative management events (blood transfusion and maybe others) [31,33-35]. The different incidence of blood transfusion within the different V_T _subgroups is difficult to explain, but it may have been more influenced by surgical technical challenge and miscalculation of blood loss/blood volume related to obesity than by a direct link to ventilatory settings. In our study, ICU admission criteria was unclear and not easily available in the electronic database, which may have also contributed to bias from a greater incidence of difficult intubation in obese patients, providers' preferences, time of day, etc. The finding of a greater incidence of pressure assisted ventilatory modes and smaller tidal volumes was not unexpected, and may reflect a greater awareness in ICU providers with the risk of conventional tidal volume ventilation. The impact of intraoperative use of large tidal volumes on the incidence of postoperative ICU admission and ALI/ARDS deserves confirmation from a multicenter study.

## Conclusions

Incidence of intraoperative ventilation with V_T _> 10 mL/kg PBW is probably underestimated because of inaccurate estimates of predicted body weight, especially in obese patients, patients of female gender or with short height. Our findings support the need of a multi-center prospective study to confirm the role of the intraoperative ventilatory insult on clinical outcomes. Meanwhile, it would be helpful to raise awareness among anesthesia providers of the risk of unintentional use of large tidal volumes in obese patients, females or patients with short height. Developing automated tools for accurate predicted body weight calculations in our electronic perioperative medical records or other interventions may help to provide the quality of ventilatory care that is intended in patients during general anesthesia.

## Competing interests

The authors declare that they have no competing interests.

## Authors' contributions

AFB contributed with study design, data collection and analysis, and manuscript drafting. CLW helped with data collection and analysis, and manuscript review. ZVT contributed with data analysis and statistical interpretation and manuscript review. PM helped with study design, data analysis, and manuscript drafting. All authors read and approved the final manuscript.

## Pre-publication history

The pre-publication history for this paper can be accessed here:

http://www.biomedcentral.com/1471-2253/11/22/prepub
